# Identification and Analysis of Regulatory Elements in Porcine Bone Morphogenetic Protein 15 Gene Promoter

**DOI:** 10.3390/ijms161025759

**Published:** 2015-10-27

**Authors:** Qianhui Wan, Yaxian Wang, Huayan Wang

**Affiliations:** Department of Animal Biotechnology, College of Veterinary Medicine, Northwest A&F University, Xi’an 712100, Shaanxi, China; E-Mails: wanqianhui_casey@163.com (Q.W.); wangyaxianwyx@163.com (Y.W.)

**Keywords:** pig, BMP15, promoter, transcription factor, LHX8

## Abstract

Bone morphogenetic protein 15 (BMP15) is secreted by the mammalian oocytes and is indispensable for ovarian follicular development, ovulation, and fertility. To determine the regulation mechanism of *BMP15* gene, the regulatory sequence of porcine *BMP15* was investigated in this study. The cloned *BMP15* promoter retains the cell-type specificity, and is activated in cells derived from ovarian tissue. The luciferase assays in combination with a series of deletion of *BMP15* promoter sequence show that the −427 to −376 bp region of *BMP15* promoter is the primary regulatory element, in which there are a number of transcription factor binding sites, including LIM homeobox 8 (LHX8), newborn ovary homeobox gene (NOBOX), and paired-like homeodomain transcription factor 1 (PITX1). Determination of tissue-specific expression reveals that LHX8, but not PITX1 and NOBOX, is exclusively expressed in pig ovary tissue and is translocated into the cell nuclei. Overexpression of LHX8 in Chinese hamster ovary (CHO) cells could significantly promote *BMP15* promoter activation. This study confirms a key regulatory element that is located in the proximal region of *BMP15* promoter and is regulated by the LHX8 factor.

## 1. Introduction

Reproductive capability is a vital economic factor for sows, for instance the Chinese Taihu breed farrows at least five more piglets per litter than the Large White breed [1]. Many studies have revealed that oocytes can produce and secrete growth factors that facilitate follicular development [2,3]. Two growth factors, bone morphogenetic protein 15 (BMP15) and growth and differentiation factor 9 (GDF9), belonging to the transforming growth factor β (TGF-β) superfamily, are produced by oocytes [4,5]. These two factors participate in the different stages of follicular development and influence the final events of embryo maturation and ovulation [6,7]. In mouse, *Bmp15* homozygous mutant causes subfertile, showing the ovulation defects and reduction of oocytes [8]. In contrast to *Bmp15* null mice, in sheep, the naturally-occurring homozygous mutants (*FecX^I^*) that has point mutations in the coding region of *BMP15* gene causes the infertile due to an arrest at the primary stage of folliculogenesis. On the other hand, the heterozygous females show increased ovulation rate and multiple pregnancies. Thus, BMP15 is related to infertility and super-fertility in a dosage-sensitive manner in sheep [9,10]. Recent reports showed that the *BMP15* mRNA level in individual denuded oocytes from single-to-triple ovulation-rate species (*i.e*., sheep) was much lower than that from high ovulation-rate species (*i.e*., pig). But the ratios of BMP15:GDF9 expression levels were significantly species-specific indicating a unique mechanism by which the species-specific ovulation-rate may be regulated [11]. A tissue screening study showed that pig GDF9 is expressed not only in ovarian tissue, but also in other somatic tissues, whereas pig BMP15 is only expressed in ovary [12]. Thus, it is worth further investigation of the transcription regulation of pig *BMP15* gene.

As a member of the BMP superfamily, BMP15, also called growth and differentiation factor 9B (GDF9B), plays a vital role in ovarian follicular development, ovulation and fertility [13,14]. The initiation of *BMP15* gene expression occurs during early follicular development in either primordial or primary follicles depending on animal species [15]. After a series of post-translational modifications, BMP15 is secreted from oocytes and forms either homodimers (BMP15:BMP15) or heterodimers (BMP15:GDF9). Both dimers can then bind to serine/threonine kinase type I–II receptors on the surface of granulosa cells, which, in turn, activates the intracellular SMAD signaling pathway [7]. Functionally, BMP15 expression in the oocyte stimulates granulosa cell proliferation and inhibits the action of follicle-stimulating hormone (FSH) by suppressing the expression of FSH receptor, which is related to ovulation rate and fertility [16]. Following identification of the functions of BMP15 in the ovary, the progress has been made towards a molecular understanding of how this gene is regulated by other factors in oocytes. Several transcription factors such as paired-like homeodomain transcription factor 1 (PITX1), germ cell nuclear factor (GCNF), LIM homeobox 8 (LHX8) and transcriptional protein Yin Yang 1 (YY1) can regulate mouse and human *BMP15* expression [17,18,19,20], however, the regulation network of pig *BMP15* gene has not been fully investigated yet.

To understand the regulatory mechanism of pig *BMP15* gene expression, we analyzed porcine *BMP15* gene 5ʹ untranslated and promoter regions, screened the primary regulatory sequence, and verified the potential transcription factors that could upregulate the expression of porcine *BMP15*.

## 2. Results

### 2.1. Molecular Cloning and Bioinformatics Analysis of Bone Morphogenetic Protein 15 (BMP15) Promoter

**Figure 1:**
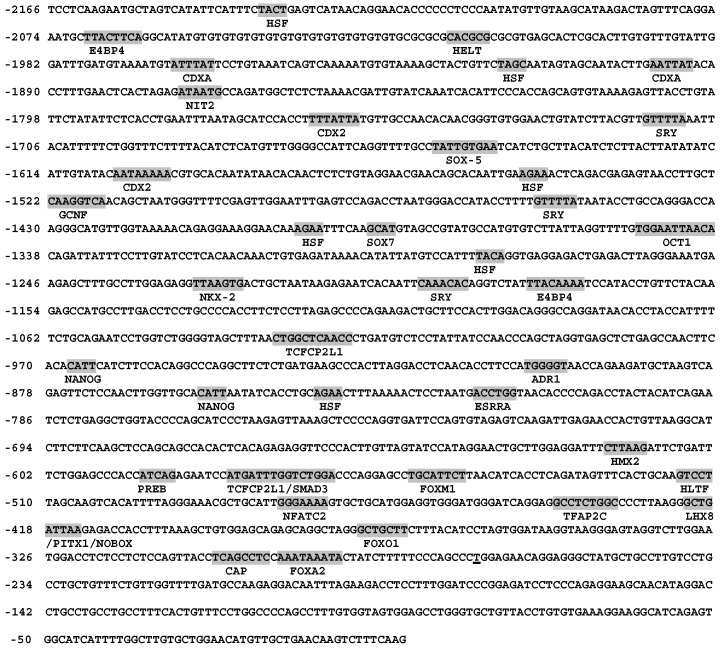
Porcine *BMP15* gene 5ʹ untranslated region (UTR) and transcription regulatory region. The 2166 bp 5ʹ UTR and promoter region of porcine *BMP15* gene was cloned from porcine ovary tissue. The potential transcription initiation site is underlined. The predicted DNA binding sites are highlighted.

**Figure 2:**
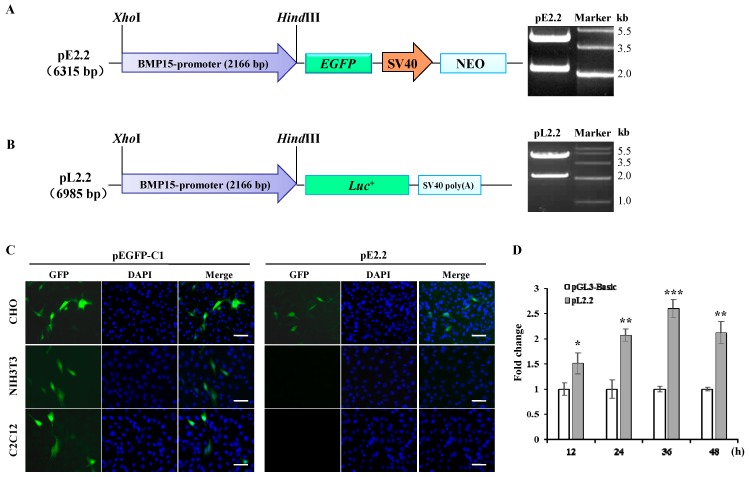
Functional analysis of cloned porcine *BMP15* promoter. (**A**) Reporter vector pE2.2 is derived from pEGFP-1 vector and contains 2.2 kb *BMP15* promoter fragment that is confirmed by Xho*I*/Hind*III* digestions; (**B**) Reporter vector pL2.2 is derived from pGL3-basic vector and contains 2.2 kb fragment confirmed by Xho*I*/Hind*III* digestions; (**C**) Cell-specific activation of *BMP15* promoter in different cell lines. Scale bar, 100 μm; and (**D**) Time-dependent luciferase assay for *BMP15* promoter activity in CHO cells. The pGL3-Basic vector was used as control. Values are represented as mean ± SD, * *p* < 0.05, ** *p* < 0.01, *** *p* < 0.001, *n* = 3.

Bioinformatics analysis showed that several potential binding sites for the reproduction related factors, including germ cell nuclear factor (GCNF), LIM homeobox 8 (LHX8), newborn ovary homeobox gene (NOBOX), and paired-like homeodomain transcription factor 1 (PITX1), were found in the promoter sequence of *BMP15* gene, indicating that BMP15 may be a downstream target of those transcription factors (Figure 1). We also found that there was no typical TATA box element in the proximal region of porcine *BMP15* promoter. Indeed, a typical core promoter element such as the TATA box does not always exist in a basic promoter region [21]. To further investigate the expression specificity of *BMP15*, a 2.2 kb promoter fragment (Accession No: KF114861.1) was subcloned into pEGFP-1 and pGL3-basic plasmid to make the reporter vector pE2.2 and pL2.2 (Figure 2A,B). The transient transfection of pL2.2 and GFP fluorescence detection showed that the *BMP15* promoter was only activated in Chinese hamster ovary (CHO) cells, but not in C2C12 and NIH3T3 cells (Figure 2C), indicating that the cloned *BMP15* promoter retained the cell-type specificity. The luciferase assays reveal the time-dependent activation of the *BMP15* promoter (Figure 2D).

### 2.2. Investigation of Primary Regulatory Elements in BMP15 Promoter

**Figure 3:**
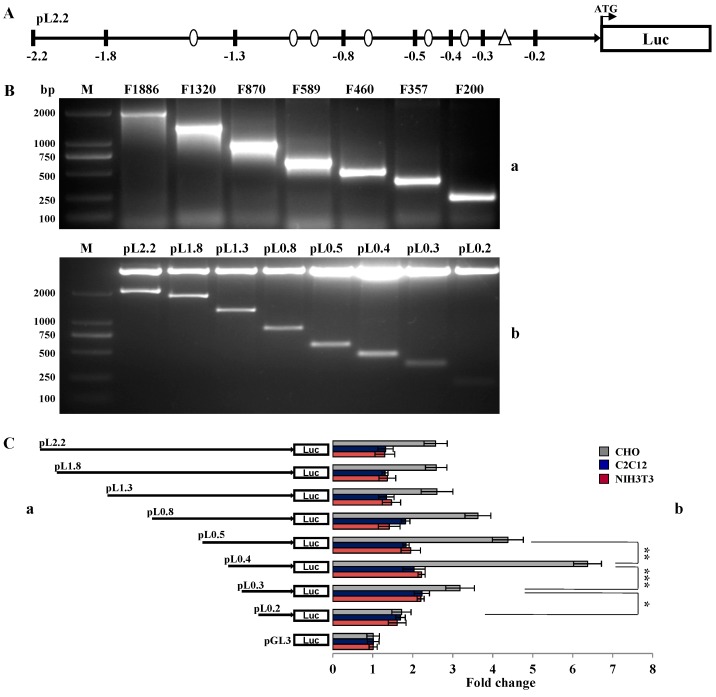
Screening of the core regulatory sequence in *BMP15* promoter. (**A**) The schematic diagram of vector pL2.2. The sites of truncated constructs are denoted in black bars. Circle dots indicate the potential DNA binding sites, and a triangle indicates the predicted transcription start site of porcine *BMP15*; (**B**) PCR amplified fragments of truncated *BMP15* promoter (**a**) and the constructs digested by Xho*I* and Hind*III* (**b**). M, 2000 bp DNA marker; and (**C**) Luciferase assays of the truncated constructs in CHO, C2C12, and NIH3T3 cells. The pGL3-Basic vector was used as control. Values are represented as mean ± SD, * *p* < 0.05, ** *p* < 0.01, *** *p* < 0.001, *n* = 3.

The procedures of promoter deletion and protein-DNA binding assays have been used to identify the essential promoter sequences [22,23]. In this study, we used the similar strategy to investigate the primary regulatory sequence of *BMP15* promoter (Figure 3A). The truncated *BMP15* promoter fragments (−1886~+4, −1320~+4, −870~+4, −589~+4, −460~+4, −357~+4, and −200~+4) were amplified from pL2.2, and were then inserted into pGL3-basic plasmid to construct reporter vectors pL1.8, pL1.3, pL0.8, pL0.5, pL0.4, pL0.3 and pL0.2 (Table S1). These recombinant plasmids were confirmed by Xho*I* and Hind*III* double digestions (Figure 3B). The truncated constructs were transiently transfected into C2C12, NIH3T3 and CHO cells, and the activity of these different *BMP15* promoter constructs was determined by a dual-luciferase assay. Results showed that BMP15 only expressed in CHO cells, but not in C2C12 or NIH3T3. The promoter activity in pL0.4 was significantly increased comparing to pL0.3 and pL0.5, suggesting that within the region of −357 to −460 bp existed an important positive regulatory element. In addition, we noticed that the BMP15 promoter activity was significantly reduced in pL0.5 *versus* pL0.4, and expected that this region contains a negative regulatory element that is worth investigating in the future (Figure 3C).

**Figure 4:**
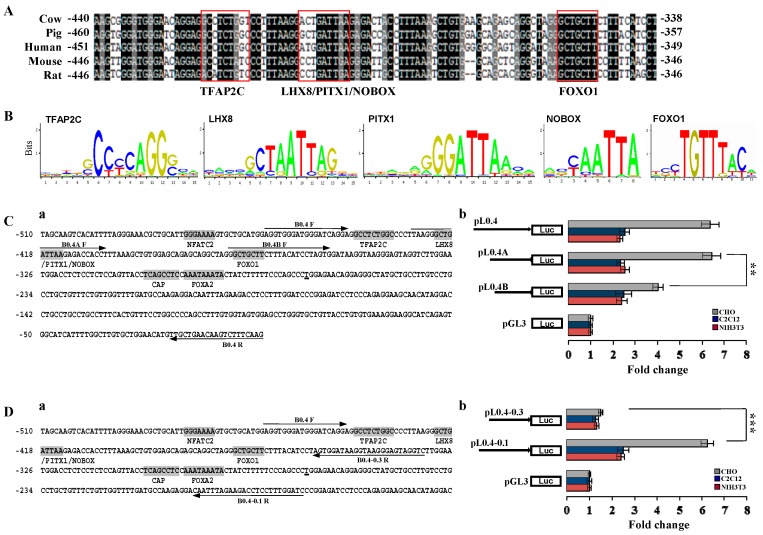
Functional analysis of regulatory elements in porcine *BMP15* promoter. (**A**) Alignment of *BMP15* promoter sequences among animal species. The potential DNA binding sites are highlighted in boxes; (**B**) The consensus sequences of TFAP2C, LHX8, PITX1, NOBOX, and FOXO1 are predicted by JASPAR program (jaspar.genereg.net); (**C**) The proximal sequence (–460 to –1 bp) of *BMP15* promoter has multiple potential DNA binding sites (**a**). Luciferase assays were performed with different deletion constructs in CHO, C2C12, and NIH3T3 cells (**b**); and (**D**) Partial proximal sequence (−460 to −175 bp) of *BMP15* promoter region (**a**). Luciferase assays were performed with different deletion constructs in CHO, C2C12, and NIH3T3 cells (**b**). The primer sequences used to generate the deletion are indicated by arrows. Values are represented as mean ± SD; ** *p* < 0.01; *** *p* < 0.001; *n* = 3.

The alignment of the DNA fragment between −357 to −460 bp region showed that this sequence was highly conserved among animal species and contained several potential DNA binding sites, including TFAP2C, LHX8/PITX1/NOBOX, and FOXO1 (Figure 4A). The consensus sequences of LHX8, PITX1, NOBOX, and FOXO1 were predicted by JASPAR program (jaspar.genereg.net) (Figure 4B). Two additional truncated constructs pL0.4A and pL0.4B were generated based on pL0.4 vector, in which the predicted TFAP2C site and LHX8/PITX1/NOBOX site was deleted, respectively (Figure 4Ca). Luciferase assays showed that TFAP2C site in pL0.4 and FOXO1 site in pL0.4B are not essential for maintaining BMP15 promoter activity, but LHX8/PITX1/NOBOX site in pL0.4A is a primary site that significantly affected the activity, because the deletion of LHX8/PITX1/NOBOX site can abolish *BMP15* activity (Figure 4Cb).

To investigate if the proximal promoter sequence could influences *BMP15* activity, two relevant reporter vectors, pL0.4–0.1 that retains the partial 5ʹ UTR sequence and CAP and FOXA2 sites (−460 to −175 bp) and pL0.4–0.3 that has no CAP and FOXA2 sites (−460 to −332 bp), were constructed (Figure 4Da). Luciferase assays showed that partially removing 5ʹ UTR sequence in pL0.4–0.1 did not affect *BMP15* promoter activity; however, the deletion of CAP and FOXA2 sites pL0.4–0.3 significantly eliminated *BMP15* promoter activity (Figure 4Db). These results indicate that the transcription initiation site exists in between −332 to −175 bp and this area is essential for *BMP15* activation.

### 2.3. LIM Homeobox 8 (LHX8) Regulates BMP15 Promoter Activation

**Figure 5:**
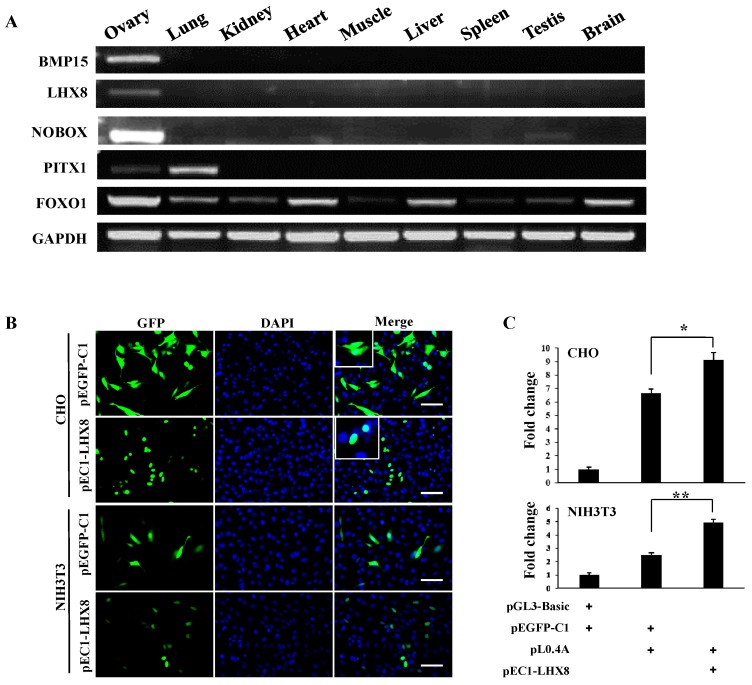
LHX8 enhances the activation of *BMP15* promoter. (**A**) RT-PCR analysis of expression of transcription factors in different porcine tissues; (**B**) Overexpression of LHX8 in CHO and NIH3T3 cells. The plasmid pEGFP-C1 was used as control. Scale bar, 100 μm; and (**C**) Luciferase assays of *BMP15* promoter activity that was enhanced by LHX8 were performed by cotransfection of vectors into CHO and NIH3T3 cells for 36 h. +, indicats a vector was transfected into the recipient cells. Values are represented as mean ± SD. * *p* < 0.05; ** *p* < 0.01; *n* = 3.

The previous reports have showed that LHX8, NOBOX, and PITX1 are critical factors for the maintenance of oocytes and folliculogenesis [18,24,25]. To investigate whether these genes were expressed in porcine ovary, we prepared mRNA samples from nine porcine tissues and performed the RT-PCR. Results showed that both *LHX8* and *BMP15* genes highly expressed in ovary tissue, but were undetectable in eight other tissues; *PITX1* was expressed in ovary and lung; and *NOBOX* in ovary and testis. The expression of *FOXO1*, a gene that plays a wide range of roles in metabolism, cell cycle and tissue differentiation, for instance myogenic growth and differentiation [26,27], was detected in all somatic tissues (Figure 5A). Thus, we cloned and constructed the *LHX8* expression vector pEC1-LHX8. The GFP-LHX8 fusion protein was observed in cellular nuclei, indicating that GFP-LHX8 fusion protein retains the translocation ability (Figure 5B). To determine the regulatory function of LHX8, the vectors pEC1-LHX8 and pL0.4A were cotransfected into CHO and NIH3T3 cells. The activity of the *BMP15* promoter was significantly enhanced by overexpression of LHX8 (Figure 5C). This result demonstrates that LHX8 is a potential positive regulator in the upstream signaling pathway of porcine *BMP15* gene. Because LHX8 was highly expressed in porcine ovary tissue (Figure 5A) and required for oogenesis in mouse [28], we speculate that LHX8 may promote *BMP15* gene expression in porcine ovary.

## 3. Discussion

In sheep and human the mutation and deletion of BMP15 gene could affect the response to controlled ovarian hyperstimulation and the birth weight, growth rate and carcass quality of lambs [29,30,31]. Thus, this gene is crucial for the ovarian function. The regulatory function and signaling pathway of BMP15 were not well illustrated in pig. Therefore, in this study, we investigated the promoter sequence of porcine *BMP15* and demonstrated the potential regulatory elements that are able to be bound by transcription factors and enhance the BMP15 expression.

The previous studies of *in situ* hybridization and immunocytochemistry confirmed the exclusive oocyte localization of mouse Bmp15 in the time of the preovulatory LH surge. Relative oocyte expression levels of Bmp15 decreased significantly after PMSG + hCG treatment [32]. In human, the expression level of *BMP15* mRNAs, which was highly expressed in high-quality embryos, was closely associated with oocyte maturation, fertilization, embryo quality, and pregnancy outcome [33]. Our previous study of *BMP15* expression in different porcine tissues (ovary, lung, kidney, heart, muscle, liver, spleen, testis, and brain) also showed that *BMP15* was only expressed in ovary [12]. To investigate the expression regulation of porcine *BMP15* gene, we constructed the porcine *BMP15* reporter vectors pE2.2 and pL2.2. Since a porcine ovarian cell line is not available and the primary-cultured ovarian cells are difficult to be obtained and handled, we, alternatively, used a CHO cell line to perform BMP15 assays using pE2.2 and pL2.2. The CHO cell line was derived from the ovary of the Chinese hamster, though it is an immortalized cell line that may not physiologically represent the ovarian epithelia, CHO cells were used to study *Bmp15* promoter activation and signaling pathways [17,34]. The results of promoter activation confirmed that the porcine *BMP15* promoter was exclusively activated in CHO cells, but not in myoblasts and fibroblasts, indicating that the cloned *BMP15* promoter retains the cell-type specificity.

Based on bioinformatics analysis and promoter deletion analysis of this study, we found that within the −427 to −376 bp region of the pig *BMP15* promoter there exists sites for transcription factors LHX8, NOBOX, and PITX1, which are the potential positive regulators to activate pig *BMP15*. Although *LHX8*, *NOBOX*, *BMP15,* and *GDF9* are highly expressed during ovarian follicle development [25,35], the definite regulatory network among these genes is under investigation. Several studies have reported an association between the BMP15 and premature ovarian failure, showing that the variant *BMP15 c.-9C>G* results in modification of its binding sites for PITX1 that trans-activates BMP15 promoter and increases the recruitment of an elevated number of follicles/oocytes early in a woman’s life [18,30,36]. Recent report showed that NOBOX was capable of up-regulating the *GDF9* promoter during human fetal ovary development [37]. *Nobox* is a downstream target gene of SOHLH1 because in *Sohlh1* null oocytes *Nobox* expression is defective resulting in a loss of genes downstream of the NOBOX pathway, such as *Gdf9* and *Oct4* [28]. *Lhx8* is also a direct transcriptional target of SOHLH1. In *Lhx8*^−/−^ mouse, ovaries fail to maintain the primordial follicles, and the transition from primordial to growing follicles does not occur, in which the expression of oocyte-specific genes, such as *Gdf9*, *Bmp15* and *Oct4*, are significantly reduced [25]. In *Lhx8* conditional knockout (cKO) oocytes, Lhx8 represses Lin28a expression indirectly interacting with the PI3K-AKT pathway and blocking the primary to secondary follicle transition [38]. On the other hand, in *Yy1*, a multifaceted polycomb group gene, cKO oocytes exhibit reduced expressions of *Lhx8*, *Gdf9* and *Bmp15*, indicating that Yy1 function is required at each of these genes to act as an activator [20]. However, the genes downstream of LHX8 in the oocyte are still unclear [28]. We in this study demonstrated that LHX8 could significantly promote the activity of pig *BMP15*, indicating that LHX8 is an upstream positive factor of BMP15. Further investigation is needed to verify whether LHX8 can directly bind to *BMP15* promoter and play a role in the regulation of folliculogenesis and granulosa cell growth in pig.

In addition, we found that the porcine *BMP15* promoter did not have the typical TATA box sequence. The results of promoter deletion analysis indicated that the sequence within −332 to −175 bp region had a CAP site (−303) and a transcription start site for pig *BMP15* gene, which was consistent with the transcription start site of rat *BMP15* [39,40].

In conclusion, a key consensus sequence is identified within −427 to −376 bp region in porcine *BMP15* promoter. The transcription factor LHX8 can specifically enhance *BMP15* promoter activation. Understanding of LHX8 to BMP15 transactivation signaling can facilitate *in vitro* maturation of porcine oocytes, which are widely used for porcine cloning, because BMP15 treatment *in vitro* could promote porcine oocyte maturation and cumulus expansion [5]. In the future, the electrophoretic mobility shift assay (EMSA) and mRNA knock-down assay may be applied to directly prove the LHX8 regulatory function.

## 4. Experimental Methods

### 4.1. Cell Culture

Chinese hamster ovary (CHO) cells were grown in DMEM/F-12 medium (Gibco, Grand Island, NY, USA) with 10% fetal bovine serum (FBS, HyClone, Logan, UT, USA). NIH3T3 fibroblasts and C2C12 myoblasts were cultured in DMEM (Gibco) with 10% FBS. All these cells were growing at 37 °C, 5% CO_2_ in a humidified atmosphere and the media were changed every 2 days.

### 4.2. Gene Cloning and Vector Construction

To construct luciferase reporter vector, a 2166 bp (ENSSSCG00000012310) DNA fragment from pE2.2 vector that was constructed by this laboratory as described previously [12] was subcloned into pGL3-basic (Promega, Madison, WI, USA) at sites of Xho*I* and Hind*III* and formed the reporter vector pL2.2. A series of truncated DNA fragments from 2.2 kb sequence were listed in Table S1 and used to construct reporter vectors that were then used to screen primary regulatory sequences of porcine BMP15 promoter.

The coding DNA sequence (CDS) of LIM homeobox 8 gene (*LHX8*) was cloned from porcine ovary, inserted into the pGEM-T Easy Vector (Promega), and confirmed by DNA sequencing. *LHX8* CDS was then subcloned into Xho*I*/BamH*I* sites of pEGFP-C1 (Invitrogen, Camarillo, CA, USA) to generate pEC1-LHX8 that could express GFP-LHX8 fusion protein.

### 4.3. Reverse Transcription-Polymerase Chain Reaction (RT-PCR)

Total RNAs were extracted from porcine ovaries by TRIzol (Invitrogen) according to the manufacturer’s instructions. The total RNAs were examined by detecting the OD260/280 ratio and the RNAs with a ratio of 2.0 were then used for reverse transcription. One microgram RNAs were reverse transcribed using oligo-dT primers, reverse inhibitor, reverse transcriptase and RNA-free water (Thermo Scientific, Waltham, MA, USA). PCR reactions using PhantaTM Super-Fidelity DNA Polymerase kit (Vazyme, Nanjing, China) in a 50 µL reaction volume were performed for 35 cycles at 94 °C 30 s, 60 °C 30 s, and 72 °C 40 s. The products of RCR were separated by 1.5% agarose gel electrophoresis. GAPDH was used as internal reference. Primers used in this study were listed in Table S2.

### 4.4. Biological Assays

To verify the tissue specificity of porcine *BMP15* promoter, 2 × 10^4^ cells were seeded in a 48-well plate 24 h before transfection. When these cells reached 80% confluence, the plasmids of pL2.2 and the control pRL-TK (10 ng) were cotransfected into CHO, NIH3T3, and C2C12 cells, respectively, by Lipofectamine 2000 Reagent (Invitrogen). For GFP fluorescence assay, the pE2.2 vector was transfected into cells for 48 h, and then the cells were fixed by 4% paraformaldehyde in PBS for 10 min. After washing 3 times with PBS, nuclei were stained with DAPI (Sigma-Aldrich, St. Louis, MO, USA), and the fluorescence images were documented with the fluorescence microscope (Nikon, Tokyo, Japan). To screen the primary regulatory sequence of BMP15 promoter, the truncated constructs, including pL1.8, pL1.3, pL0.8, pL0.5, pL0.4, pL0.3 and pL0.2, were transfected into CHO, C2C12 and NIH3T3 cells, respectively. In order to determine whether the GFP-LHX8 fusion protein located in cellular nuclei, pEC1-LHX8 and pEGFP-C1 was transfected into CHO and 3T3 cells for 48 h, and then the cells were fixed by 4% paraformaldehyde in PBS for 10 min. After washing 3 times with PBS, nuclei were stained with DAPI and the fluorescence images were documented with the fluorescence microscope. To verify the interaction of LHX8 with the *BMP15* promoter, pL0.4A construct was cotransfected with pEC1-LHX8 into CHO and 3T3 cells, respectively.

For dual-luciferase assays, cells were harvested at 36 h after liposome-mediated transfection and lysed for 15 min at room temperature. Luciferase activity was stimulated by adding luciferase assay reagents (Promega) and then detected by a BHP9504 Luminometer (Hamamatsu, Japan). The average ratio of firefly luciferase light units to Renilla luciferase light units was calculated and we conducted three independent experiments and then collected the data for analysis. Statistical significance was accepted at *p* < 0.05 and determined using one-way ANOVA followed by post-hoc tests (Newman–Keuls method).

### 4.5. Bioinformatics Analysis

The online programs of MatInspector function of Genomatix Suite (www.genomatix.de), JASPAR (www.jaspar.genereg.net), and Gene Regulation [41] were used to predict the potential primary regulatory region and transcription factor binding sites on *BMP15* promoter (−2166 to −1 bp). ClustalW2 software (http://www.ebi.ac.uk) was used for multiple alignments of DNA sequences from different animal species (Cow, ENSBTAG00000045782; Pig, ENSSSCG00000012310; Human, ENSG00000130385; Mouse, ENSMUSG00000023279; Rat, ENSRNOG00000002984).

### 4.6. Statistical Analysis

One-way ANOVA was used and Newman–Keuls multiple range tests were conducted as post-hoc tests if a significant difference (*p* < 0.05) existed in a group of data. Values were represented as the mean ± SD, and statistical significance was indicated as follows: * *p* < 0.05; ** *p* < 0.01; *** *p* < 0.001. Data were representative of at least three independent experiments.

## Supplementary Materials

Supplementary materials can be found at http://www.mdpi.com/1422-0067/16/10/25759/s1.
