# Supplementary material for: Identification and Analysis of Regulatory Elements in Porcine Bone Morphogenetic Protein 15 Gene Promoter
Source: Int J Mol Sci. 2015 Oct 27;16(10):25759–72. doi: 10.3390/ijms161025759 (PMC4632825; doi:10.3390/ijms161025759)
Supplement: Supplementary file 1 [file ijms-16-25759-s001.pdf]

Supplementary Information

Table S1. Vectors used in this study.

Name	Vector	Inserted Fragment Name	Inserted Fragment Size (bp)
pE2.2	pEGFP-1	–	2166
pL2.2	pGL3-Basic	F2166	2166
pL1.8	pGL3-Basic	F1886	1886
pL1.3	pGL3-Basic	F1320	1320
pL0.8	pGL3-Basic	F870	870
pL0.5	pGL3-Basic	F589	589
pL0.4	pGL3-Basic	F460	460
pL0.3	pGL3-Basic	F357	357
pL0.2	pGL3-Basic	F200	200
pL0.4A	pGL3-Basic	F427	427
pL0.4B	pGL3-Basic	F376	376
pL0.4-0.3	pGL3-Basic	F128	128
pL0.4-0.1	pGL3-Basic	F285	285
pEC1-LHX8	pEGFP-C1	*LHX8*	888
pEGFP-C1	pEGFP-C1	–	–

Table S2. Primers used in this study.

Name	Sequences	Accession Number	Size (bp)
B2.2	F: TAGCAAAATAGGCTGTCCCC<br>R: CCCAAGCTTCCATCTTGAAAGACTTGTTCAGCAA [a]	KF114861.1	2166
B1.8	F: CCGCTCGAGTGAACTCACTAGAGATAATGCCAGA<br>R: CCCAAGCTTCCATCTTGAAAGACTTGTTCAGCAA	KF114861.1	1886
B1.3	F: CCGCTCGAGCCTCACAACAAACTGTGAGATAAAA<br>R: CCCAAGCTTCCATCTTGAAAGACTTGTTCAGCAA	KF114861.1	1320
B0.8	F: CCGCTCGAGCAACTTGGTTGCACATTAATATCAC<br>R: CCCAAGCTTCCATCTTGAAAGACTTGTTCAGCAA	KF114861.1	870
B0.5	F: CCGCTCGAGATCAGAGAATCCATGATTTGGTCTG<br>R: CCCAAGCTTCCATCTTGAAAGACTTGTTCAGCAA	KF114861.1	589
B0.4	F: CCGCTCGAGGTGGGATGGGATCAGGA<br>R: CCCAAGCTTCCATCTTGAAAGACTTGTTCAGCAA	KF114861.1	460
B0.3	F: CCGCTCGAGTGGATAAGGTAAGGGAGTAGGTC<br>R: CCCAAGCTTCCATCTTGAAAGACTTGTTCAGCAA	KF114861.1	357
B0.2	F: CCGCTCGAGCAATTTAGAAGACCTCCTTTGGATC<br>R: CCCAAGCTTCCATCTTGAAAGACTTGTTCAGCAA	KF114861.1	200
B0.4A	F: CCGCTCGAGTAAGGGCTGATTAAGAGACCACCT<br>R: CCCAAGCTTCCATCTTGAAAGACTTGTTCAGCAA	KF114861.1	427
B0.4B	F: CCGCTCGAGGGCTGCTTCTTTACATCCTAGTG<br>R: CCCAAGCTTCCATCTTGAAAGACTTGTTCAGCAA	KF114861.1	376
B0.4-0.3	F: CCGCTCGAGGTGGGATGGGATCAGGA<br>R: CCCAAGCTTGACCTACTCCCTTACCTTATCCACT	KF114861.1	128
B0.4-0.1	F: CCGCTCGAGGTGGGATGGGATCAGGA<br>R: CCCAAGCTTGATCCAAAGGAGGTCTTCTAAATTG	KF114861.1	285

Table S2. *Cont.*

Name	Sequences	Accession Number	Size (bp)
GAPDH	F: ACCTGCCGCCTGGAGAAACC<br>R: GACCATGAGGTCCACCACCCTG	NM_001206359.1	252
BMP15	F: TGGTCCTCCTCAGCATCATTAG<br>R: TAGGTGAAGTTGATGGCGATAAA	NM_001005155.1	428
FOXO1	F: CCTACTTCAAGGATAAGGGCGA<br>R: GGTGGGTACACCATAGAATGCA	NM_214014.2	466
LHX8	F: CTGTGCTGGCATGTTCGGT<br>R: GGGCACCTTCAACACTTATTCC	NM_001166315.2	361
LHX8 CDS	F: CCG<u>CTCGAG</u>CCATGGCCTCGGGCTCCGTCT<br>R: CGC<u>GGATCC</u>TTAGGTATGACTTATTGGCAGTTG	NM_001166315.2	888
NOBOX	F: CAGCCCCAGTTTCCATACCT<br>R: CCAAAGCCTGAGCATACGG	NM_001195116.1	319
PITX1	F: CAACGCACGCACTTCACAAG<br>R: GAGTTAAGCGAGGAGCCAGTGA	NM_001244370.1	491

[a] AAGCTT, CTCGAG, GGATCC refer to restriction enzyme sites of Hind*III*, Xho*I* and BamH*I*.
